# Filling of carbon nanotubes and nanofibres

**DOI:** 10.3762/bjnano.6.53

**Published:** 2015-02-19

**Authors:** Reece D Gately, Marc in het Panhuis

**Affiliations:** 1 Soft Materials Group, School of Chemistry, University of Wollongong, Wollongong, NSW 2522, Australia; 2 Intelligent Polymer Research Institute, ARC Centre of Excellence for Electromaterials Science, AIIM Facility, University of Wollongong, Wollongong, NSW 2522, Australia

**Keywords:** applications, carbon nanostructures, filling, nanofibers, nanotubes

## Abstract

The reliable production of carbon nanotubes and nanofibres is a relatively new development, and due to their unique structure, there has been much interest in filling their hollow interiors. In this review, we provide an overview of the most common approaches for filling these carbon nanostructures. We highlight that filled carbon nanostructures are an emerging material for biomedical applications.

## Introduction

Carbon nanotubes are well-known, 1D nanostructures, which are comprised of single, double or multiple coaxial layers of graphene [1]. The synthesis method and conditions greatly affect their structural characteristics such as number of layers, length and diameter distribution [2]. For example, the arc discharge synthesis method can be used to produce multiwalled carbon nanotubes (MWCNTs) with an inside diameter as small as 0.4 nm [3] and an outside diameter of up to 200 nm [4]. It has been shown that the inside diameter of MWCNTs produced using chemical vapour deposition is proportional to the size of the metal catalyst used during production [5]. Their high specific surface area (SSA, 1315 m^2^/g) [6] makes MWCNTs an ideal material for application in hydrogen storage [7], capacitors [8] and sensing [9].

Single-walled carbon nanotubes (SWCNTs) were discovered during investigations into the filling of MWCNTs with iron and cobalt [10–11]. Rather than producing a filled MWCNT, the metals acted as a catalyst to create a nanotube with a single wall. Similar to MWCNTs, the physical properties (inside diameter, length, degree of graphitization) of SWCNTs vary with the production method [12]. For example, during the synthesis of SWCNTs using pulsed laser vaporization (PLV), the temperature of the deposition chamber was used to influence the average inside diameter range. It was demonstrated that at 780 °C the average diameter was 1.0 nm, whereas at 1000 °C the average diameter increased to 1.2 nm [13]. PLV has also been used to produce large quantities of pure SWCNTs and MWCNTs [14]. Other research showed that applying a magnetic field had a large effect on the size of the SWCNTs [15–18]. The SSA of the SWCNTs can be between 124 and 1024 m^2^/g depending on the production method used by the various manufacturers [19]. Arc discharge has also been used to produce bundles of SWCNTs with a yield of up to 90% [20]. This technique is now one of the more common production techniques, in addition to catalytic carbon vapour deposition (CCVD).

Carbon nanofibres (CNFs) are larger (≈100 nm outer diameter), cylindrical, carbon structures that have multiple possible structures. In order to ease the complexity that surrounds all of these possible structures, the nomenclature proposed by Suares-Martinez et al. shall be used for the most part [21]. The structures that have been observed include: hollow- and filled-core, stacked nanocones; partitioned, stacked nanocones; and partitioned nanotubes. The filled-core, stacked nanocone structure consists of bowed sheets of graphene, which are stacked to produce a cylindrical, solid structure [22–23]. As this produces solid structures (and thus not applicable as a material to be filled), this review will focus on those produced by catalytic thermal chemical vapour deposition using a floating catalyst, henceforth referred to as vapour-grown CNFs (VGCNFs) [24]. This technique can be used to produce partitioned, stacked nanocones or partitioned nanotubes. VGCNFs are produced (depending on the structure of the catalyst) with a hollow interior cavity [25] and have been shown to consist of two primary structures, single layer and double layer. The internal arrangement is the same for both structures, that is, a series of parallel graphitic layers at an angle of 4–36° relative to the hollow core. The double layer CNFs have an additional second layer (outside the angled layer) consisting of multiple sheets of graphene, which are aligned parallel with the core [12,26]. Since it has been shown that thermal treatment between 1300 °C and 1700 °C leads to improved electrical and mechanical properties, VGCNFs are typically heat treated [27]. Typical dimensions of CNFs are: outside diameters of up to 200 nm, inside diameters of 12.5 nm (single layer) or 22 nm (double layer), and lengths of up to 20 µm [24,26]. The SSA depends on the degree of heat treatment. For example, a SSA of 37 m^2^/g results from heat treatment at 1200 °C and is reduced to 15 m^2^/g after heat treatment at 2800 °C. Pyrolitic stripping can also be performed on as-grown nanofibres to remove unreacted polyaromatic hydrocarbons that may have fused onto the surface of the VGCNFs. This has been shown to increase the SSA from 20 m^2^/g to 62 m^2^/g [28]. Even higher values (348 m^2^/g) have been reported when using a metal catalyst and C_3_H_8_ decomposition [29]. Due to their unique internal structure, there has been significant interest in the filling of CNFs for the alignment of atoms [30–31].

**Figure 1:**
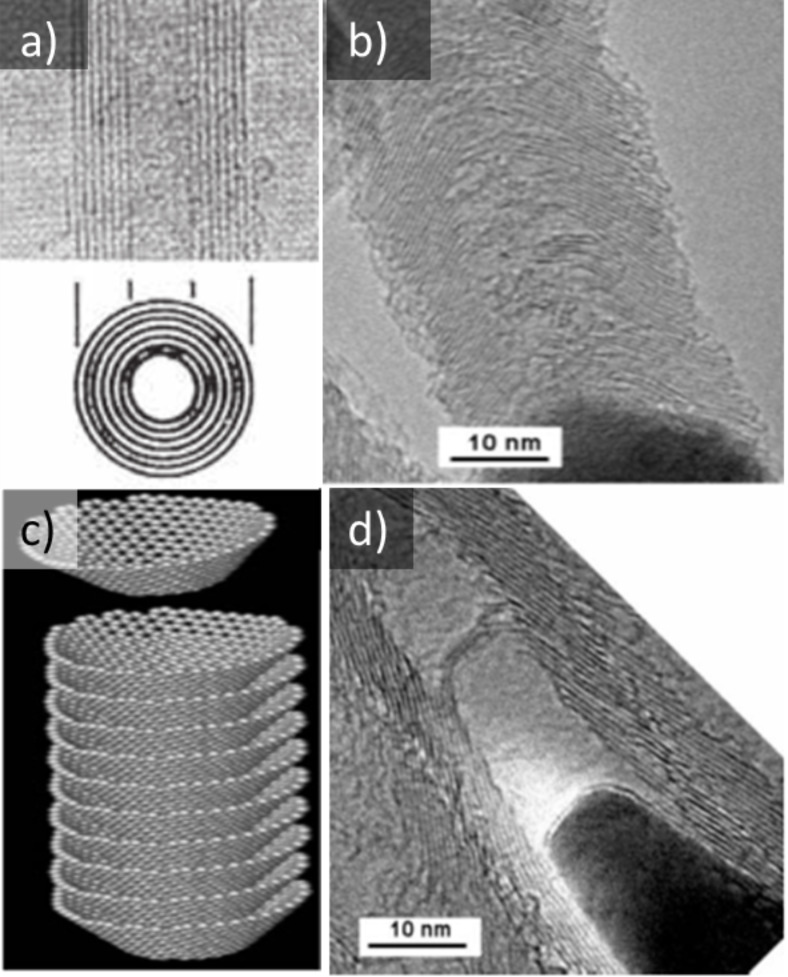
Images of (a) a typical MWCNT and (b,d) typical VGCNFs. A schematic representation is given in (c) of the “cup-stacked” VGCNFs, resulting in a hollow core. (a) Adapted with permission from [3], copyright 2006 the Royal Society of Chemistry and (b,c,d) with permission from [23], copyright 2007 the Royal Society of Chemistry.

Figure 1 shows the morphological differences between a typical MWCNT and a typical VGCNF. Due to their large (average) inside radius, VGCNFs have a larger inside area as compared to SWCNTs and MWCNTs of the same length. The main interest in the filling of VGCNFs comes from their unique angled structure: the slight angle produces internal “shelves”. This unique structure is clearly visible in the microscopy image (Figure 1b,d) and the schematic representation (Figure 1c).

The filling of these specific tubular carbon nanostructures (TCNSs) has attracted much interest due to their applications in gas storage (in particular H_2_) [32–33], electrochemical energy storage [34–35], battery electrodes [24,36], catalysis [37–38] and nanowelding [39]. Shortly after their discovery in 1991, MWCNTs were filled with metals in order to create metal nanowires encapsulated within the CNT [10]. While this was the original inspiration behind the filling of TCNSs, it is still very much prevalent today. The main advantages of using CNTs to produce metallic nanowires is that the CNTs act as a template for self-assembly of the nanowires [40–41] and the CNT structure can act as a protective sheath to protect the nanowire from being damaged by chemicals in harsh conditions/environments [42]. CNTs have also been used as sacrificial templates. The CNTs can be removed by heat treating (generally requiring temperatures greater than 600 °C), thereby leaving the metal structures unaltered and exposed [43–44]. It has also been shown that the irradiation of a filled MWCNT with a focused, high energy (200 keV) electron beam results in MWCNT–Co–MWCNT junction sites [45–46].

There has been considerable interest in the filling of SWCNTs and MWCNTs for drug delivery applications, for example, tip functionalization of the filled CNT for selective drug release [47–48]. A recent review focussed on the use of CNTs filled with antitumour medication for use in chemotherapy and immunotherapy [49]. In particular, they noted that the high level of selectivity (when functionalized) gave the CNTs the ability to “seek out” and selectively deliver the contained drugs to the tumours. The same article also discussed the potential use of SWCNTs for the treatment of central nervous system disorders due to their ability to pass through blood–brain barriers [50].

In this article, selected avenues for the filling of carbon nanotubes and nanofibres as well as applications of the filled TCNSs (including biomedical and catalytic applications) are discussed. We will present the progression of selected TCNS filling techniques beginning from the earliest papers that can be found as well as how the techniques have adapted to modern methods and applications, and finally reviewing some of the most recent papers that have been released. The most common ex situ techniques for opening (decapping the TCNSs) and filling are also described in detail, as this is one of the main problems to overcome when filling TCNSs. The unique advantages with regards to the filling of these two TCNSs are also discussed in this review, as it is important to emphasize that both serve their own purposes.

## Review

### Approaches for filling carbon nanostructures

#### Opening the capped ends

It is well known that the as-produced TCNSs have one end capped [51], thus in order to achieve filling, the TCNSs must be opened. The initial work focussed on the opening of MWCNTs using bismuth in the presence of oxygen at 850 °C [52]. This was then further expanded by suspending the MWCNTs in 68% nitric acid and refluxing at 140 °C for 4.5 h [53]. This wet chemistry method of opening MWCNTs was also attempted on SWCNTs using a heated hydrochloric acid solution, as nitric acid was found to be too harsh for SWCNTs. Similar to the MWCNTs, the SWCNTs were then easily filled directly after opening [54]. This was followed by more sophisticated methods such as oxygen plasma treatment [55], electrochemical treatment (which was able to remove the caps for both SWCNTs and MWCNTs) [56], heat treatment in carbon dioxide/air [57], sonication-induced shearing [58–59], partial opening due to purification [60], precision cutting [61] and water-assisted etching [62].

The main issue with these methods is that etching the CNTs in this way damages their surface or alters their morphology in undesired ways (e.g., reduction in length). Due to this challenge, much research has been focussed on producing CNTs that are open at both ends. This has been achieved by using an anodic aluminium oxide (AAO) film as a template for the thermal decomposition of hydrocarbon gases, followed by removal of the template by etching in 46% HF solution [63–64]. This technique has since been refined to produce tailored CNTs of desired length and diameter [65] and to allow the template to be dissolved in NaOH (rather than concentrated HF) [66]. This method has also been employed to produce SWCNTs [67] and VGCNFs [68], as well as long (10 µm) MWCNTs that can be nested or joined to create long MWCNTs [69].

The three primary methods for opening TCNSs are: electrochemical filling, functionalization of the TCNSs, and a method that takes advantage of the capillary forces within the TCNSs. Although it is also possible to combine more than one technique (e.g., chemical and capillary [70]), here we describe the general principle of each method individually.

#### Electrochemical filling

Electrochemical deposition on both the interior and exterior surfaces of TCNSs has been achieved [71]. MWCNT samples decorated with gold nanoparticles on the interior and exterior surfaces were produced by first functionalizing the CNTs with carboxylic acid groups [65]. This was followed by sonication of the functionalized MWCNTs in water and application of a large (225 V) potential across two gold electrodes [72]. This was shown to not only decorate the external surface, but also to fill some MWCNTs with a gold nanowire. Ordered, open MWCNTs produced from the AAO template method have also undergone an electrochemical filling process with nickel–iron alloys [73]. It was shown that this technique could be used to control the nickel/iron ratio and the amount of filling. Electrochemical methods have also been used to fill TCNSs with water [74] and can take advantage of the presence of oxygen within TCNSs applied as an electrode for Li–O_2_ rechargeable batteries [75].

#### Filling through functionalization

The functionalization of TCNSs is a proven, effective method to coat the exterior [8,76–77] or interior [78] surface with metal particles. This method produces TCNSs with a single-atom-thick layer (plus the functional group, typically a carboxylate group) of metal particles. It has been utilised to create nanoscale capacitors with high energy storage rates, high specific capacitance (329 F/g) [8,79] in addition to high surface area electrodes [76–77,80]. TCNSs have also been used as a membrane for water and gas filtration [81]. It has been proposed that by chemically functionalizing the TCNSs, the selectivity of the membrane can be adapted to remove specific contaminants [82–83]. Tip functionalization has also been used as a method to selectively separate various analytes [84–85] and can be performed at room temperature using ozone and small amounts of water vapour [86].

#### Capillary filling

It is well known that a tubular structure with a high aspect ratio will have strong capillary forces, and this is especially true for TCNSs [87–88]. These forces can be exploited to achieve the filling of TCNSs. Capillary filling was achieved by depositing drops of metallic lead onto the external surface of MWCNTs, followed by heat treating the sample to 400 °C [89]. This heat treatment removed the capped ends and the resulting capillary action led to the filling of the MWCNT through the absorbance of the liquid lead. This process has been modelled to determine the extent of the capillary action [90]. In addition, the effect of the molecular weight of a polymer on the capillary action has also been investigated [91]. Microscopy has been used to visualize the filling of a MWCNT with gold via Joule heating and capillary action [92]. A mathematical model has been developed to evaluate the relationship between the TCNS radius, the radius of the nanoscale drop of material used to fill the TCNS, and the contact angle between the filler and the TCNS. This model successfully predicted the capillary absorption of non-wettable nanoparticles [93] and has been employed to achieve filling [94] as well as removal of the encapsulated nanoparticles [95]. Sonication has also been employed to fill MWCNTs in solution. Sonication acted to shear the MWCNT, resulting in the filling of the MWCNT with the surrounding metal solution [96].

Other methods have employed focused electron irradiation to produce SWCNTs within the core of a filled MWCNT. This was achieved by first filling a MWCNT with iron, cobalt, nickel, or an iron–cobalt alloy using capillary action. Following this step, the sample was placed inside a transmission electron microscope (with the sample stage temperature set at 600 °C) and subjected to electron irradiation [97]. This resulted in the growth of a SWCNT within the inner core of the MWCNT.

#### Vapour-phase filling

Filled SWCNTs and MWCNTs have been achieved by exposure to a metallic vapour. This resulted in metal nanowires within the core of the CNT [98–101]. A general summary of this method is as follows: the as-produced SW/MWCNTs were processed in a furnace under vacuum at a temperature that vaporizes the compound/element to be filled into the CNT. Next, a stream of the metallic vapour was sent into the furnace. In one such study, purified double-walled CNTs (DWCNTs) were added to an evacuated furnace at 400 °C and 10^−3^ Pa containing VCl_3_ vapour [101]. The resulting material was then cooled to 100 °C for 48 h and cleaned with a HF solution. This filling method not only fills the interior cavity, but also coats the exterior of the CNTs with the vapour. Therefore, an additional cleaning step is required to remove these species.

It is important to note that this filling method (along with many others mentioned in this article) is not restricted to metallic filling, although it is one of the more prevalent topics. Botka et al. have performed multiple studies on methods to efficiently fill SWCNTs with coronene [102–103]. In both cases, the filling was performed by first opening the SWCNTs, then placing them in a furnace with coronene at temperatures of up to 450 °C and at a pressure of 10^−4^ mbar – the ideal conditions for the sublimation of coronene. After processing it was found that the coronene had both coated the SWCNT, as well as filled it. In some instances, this annealing and vapour filling caused a structural change in the coronene (which formed dicoronylene, the dimer of coronene) or in the SWCNT itself (which produced a DWCNT at high temperatures). This effect along with the unique structure of CNTs was exploited to produce linear diamondoid assemblies of adamantine within the core of the DWCNTs using a method based on that proposed by Zhang et al. [104].

#### In situ filling

**Figure 2:**
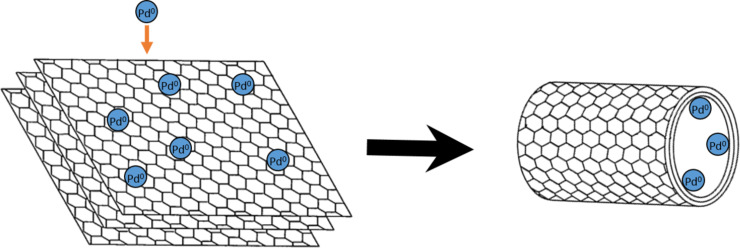
Schematic of a method for producing filled SWCNTs and MWCNTs via arc discharge in solution. Metallic palladium particles decorated the exterior surface of graphene sheets, which were then processed as filled CNTs. This schematic was inspired by a figure appearing in reference [106].

Although a significant amount of effort has gone into developing approaches for opening the end caps of TCNSs, some research has focussed on methods that either fill in situ (during growth) or that simultaneously open and fill. These methods make use of the strong capillary action that exists within the nanoscale cavities. Filled SWCNTs and MWCNTs have been produced using layered sheets of graphene, decorated with palladium particles via arc discharge in solution [105–106] (see Figure 2). This approach was able to produce filled SWCNTs or MWCNTs, depending on the number of layers of graphene used. In addition, it has been proposed that other metallic particles can be used. This has been demonstrated with Pd–Ag nanoparticles on graphene sheets, however, they were not “rolled” into CNTs [107].

Both vapour phase filling and in situ filling techniques have been combined to produce MWCNTs that are doped with various other elements, such as phosphorous and nitrogen, which can then later be released under high temperature conditions [108–109]. One method to produce nitrogen-doped MWCNTs is a modified, floating catalyst, CVD technique, which is most commonly used to produce VGCNFs. The catalyst (ferrocene) was placed into the CVD chamber under an argon/ethylene flow, and melamine was used as an efficient source for N-doping [110]. The chamber was then heated to 950 °C, with an effective heating of the ferrocene and melamine to 350 °C. At this temperature both compounds undergo sublimation. After the purification steps, it was found that the resulting MWCNTs were highly doped with nitrogen, demonstrating yet another method to produce MWCNTs to be used as a source of nitrogen storage. The nitrogen could be removed by heating to 700 °C. A very detailed review on one-dimensional nitrogen-containing carbon nanostructures by Ćirić-Marjanović et al. contains a very detailed list of other structures and methods used to achieve nitrogen storage [111].

The arc discharge method was also modified to produce filled SWCNTs and MWCNTs [112–113]. This was achieved by first producing a powdered mixture of graphite and the various metals performed in the study (YB_2_, YNi_2_, and NiB). Next, standard arc discharge methods were utilised (25–35 V, 100 A). This method produced filled MWCNTs, as well as filled, graphitic nanoparticles. This method has been employed in conjunction with an AAO template lined with the filler material [114] or completely filled with the metal [115]. Moreover, a variety of bismuth–tin nanostructures covered by CNTs have been produced via in situ filling. It was demonstrated that the MSnO_2_/MBi_2_O_3_ ratio was instrumental in the formation of the encased nanostructures (nanoparticles, nanorods, and nanowires) [116].

#### Recent developments

Although several drug delivery and medical imaging applications of SWCNT and MWCNTs have been identified, this line of research has only very recently emerged [117–119]. Further investigations into the selective binding of functional groups and various viruses or tumours could provide for an effective drug delivery system for treating various difficult-to-treat ailments. However, effective CNT filling methods that do not negatively impact the drug require further investigation. To date, only SWCNTs and MWCNTs have been evaluated for their use in drug delivery. Although potentially promising for affordable, targeted, drug release, VGCNFs have not yet been evaluated. Whilst they have not demonstrated the same nanoscale interactions as CNTs (such as crossing the blood–brain barrier, which is still under investigation), they may have other applications on the larger scale and allow for higher drug storage capacity.

The use of focused electron beam irradiation for the removal of the outside layers of SW/MWCNTs has promise as an effective method to produce pure or alloyed nanowires for conductive applications [120]. This, however, is also a relatively new research field where very little investigation into developing a scalable method for producing larger quantities of nanowires has been undertaken. As TCNSs have shown promise in the field of tissue engineering, with further development, this method may be used to produce channels for cell growth. Filled with appropriate drugs and medium, TCNSs may provide a platform for the growth of various cells.

Although significant research has gone into the filling of TCNSs, possibility for further exploration still remains. For example, most of the current methods are harsh (i.e., employing concentrated acids, high temperature/pressure conditions) and damage either the filler material or the TCNS itself. In addition, the filling process can be complex and can require multiple steps using custom-built equipment. These methods could be improved to provide a simple method to completely fill the TCNSs with the desired material, without damage to the TCNS, and in a timely manner. Simplicity is key for scale-up to large quantities of filled TCNSs, which is a requirement for any commercially viable application.

TCNSs provide a unique structure with a high aspect ratio, large filling volume, and good stability, which can be useful for many applications. For example, it is well known that they can improve the mechanical and electrical properties of various dispersants [121–124]. However, the properties of the resulting materials prepared from dispersions of TCNSs filled with various materials have not been fully investigated. One interesting application area is in the field of repair (healing) of polymer materials [106]. Traditionally crack healing, which can repair the detrimental effects of mechanical degradation and fatigue in polymer adhesives, was primarily investigated this field. One application of this concept could be in the area of polymer materials (such as hydrogels) for cartilage replacement.

**Figure 3:**
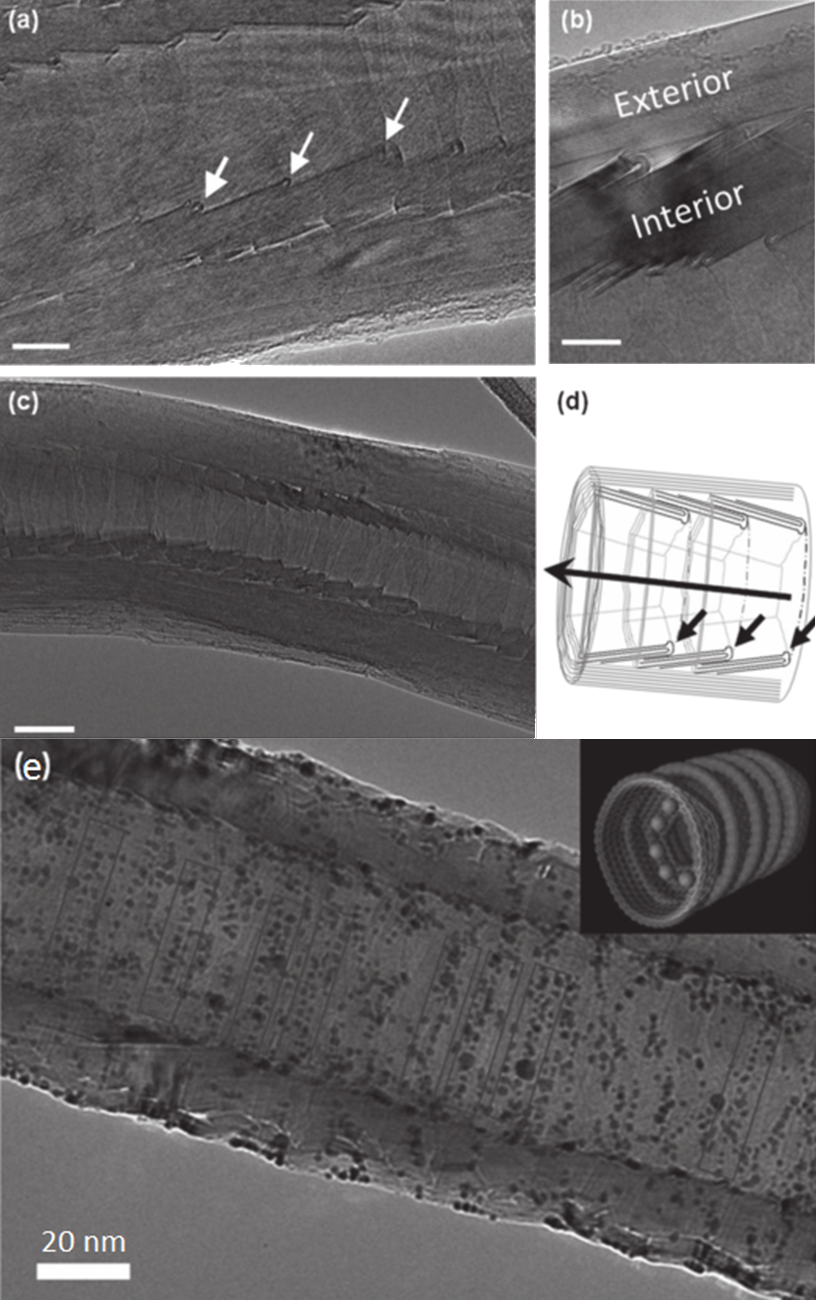
(a,b,c) Transmission electron micrographs of a hollow CVD-grown CNF with the graphene caps indicated by the white arrows. (d) Schematic of the structure of the CNFs with the caps as well as nucleation sites indicated by the black arrows. (e) Transmission electron micrograph of the filled CNFs with gold nanoparticles. Inset: a computer generated model. Reprinted with permission from [30], copyright 2012 Wiley-VCH.

Most interest in the filling of VGCNFs is due to their unique internal structure, which allows for the alignment of atoms along the ridges of the graphene sheets [38], as shown in Figure 3. The internal structure has been demonstrated to be a good site for the catalytic growth of nanoparticles of desired dimensions (Figure 3a,d). When the nanoparticles grow too large, they simply flow through and out of the VGCNF, as indicated in Figure 3d [125–126]. The structure of VGCNFs in the context of filling has not been extensively studied [127], leaving much to be discovered as to how this unique internal “layering” can assist with self-assembly and size-controlled particle growth.

## Conclusion

The filling of TCNSs has shown much promise regarding the synthesis of nanowires, hydrogen storage, and drug delivery. Although most of the research has focussed on the development of the actual methods for filling, there are a number of biomedical applications of these fascinating materials that remain to be explored and developed. In this review, we aimed to provide an overview of the most common methods for filling of SWCNTs, MWCNTs and VGCNFs. Most of the reviewed literature relates to MWCNTs, as this material has been extensively studied. VGCNFs are an emerging material for filling applications, but not all VGCNFs are suitable. For example, the VGCNFs with a “deck of cards” morphology (i.e., a series of parallel graphene sheets stacked on top of each other [128]) cannot be filled due to the lack of a hollow core [22]. SWCNTs have significant promise, however, their high production cost has limited research in the past. As research into the efficient production of pristine SWCNTs has progressed, their cost has correspondingly rapidly decreased, which should lead to future SWCNT filling applications.

This article highlighted TCNSs as a suitable material for applications requiring filled nanostructures, as well as the unique strengths of both CNTs and CNFs. The remaining challenge is to prepare filled TCNS materials that achieve one or both of the following properties: (1) selective drug delivery using nanostructures, which is important for the development of nano-sized needles or patches for the localised treatment of diseases; (2) autonomic healing of polymeric materials, such as tough hydrogels, which is important for load-bearing biomedical materials such as cartilage replacement. While there is much research into the filling of SWCNTs and MWCNTs, there is very limited research regarding the filling of VGCNFs, which have been demonstrated to be as efficient (if not more) for certain filling applications such as storage and self-assembly. However, a more reliable and thorough method to completely fill VGCNFs must be established, as current methods either result in partial filling, or can only fill with certain types of materials. In conclusion, it is clear that filled TCNSs offer great opportunities for a broad variety of applications, yet a number of challenges remain to be addressed.
